# Microchip Immunoassays for Monitoring Renal Function: Rapid, Low-Cost, and Highly Sensitive Quantification of Urinary Biomarkers of Diabetic Nephropathy

**DOI:** 10.3390/mi12111353

**Published:** 2021-10-31

**Authors:** Toshihiro Kasama, Miaomiao Sun, Noritada Kaji, Shin’ichi Akiyama, Yukio Yuzawa, Manabu Tokeshi, Seiichi Matsuo, Yoshinobu Baba

**Affiliations:** 1 Department of Bioengineering, Graduate School of Engineering, The University of Tokyo, Tokyo 113-8654, Japan; 2 Institute of Nano-Life-Systems, Institutes of Innovation for Future Society, Nagoya University, Aichi 464-8603, Japan; kaji@cstf.kyushu-u.ac.jp (N.K.); tokeshi@eng.hokudai.ac.jp (M.T.); babaymtt@chembio.nagoya-u.ac.jp (Y.B.); 3 Department of Biomolecular Engineering, Graduate School of Engineering, Nagoya University, Aichi 464-8603, Japan; tkzmtap@kyudai.jp; 4 Department of Applied Chemistry, Faculty of Engineering, Kyushu University, Fukuoka 819-0395, Japan; 5 Division of Nephrology, Department of Internal Medicine, Nagoya University Graduate School of Medicine, Aichi 466-8550, Japan; akiyama.med@gmail.com (S.A.); smatsuo@med.nagoya-u.ac.jp (S.M.); 6 Department of Nephrology, Fujita Health University, Aichi 470-1192, Japan; yukio-y@fujita-hu.ac.jp; 7 Division of Applied Chemistry, Faculty of Engineering, Hokkaido University, Hokkaido 060-0808, Japan; 8 Health Research Institute, National Institute of Advanced Industrial Science and Technology, Kagawa 761-0395, Japan

**Keywords:** immunoassay, microchannel device, microbead, biomarker, diabetic nephropathy, urine

## Abstract

This study developed low-cost and highly sensitive immunoassay devices possessing the ability to rapidly analyze urine samples. Further, they can quantitatively detect three biomarkers indicating renal injury: monocyte chemotactic protein 1 (MCP-1), angiotensinogen (AGT), and liver-type fatty acid binding protein (L-FABP). The devices were used to successfully estimate the concentrations of the three biomarkers in urine samples within 2 min; the results were consistent with those obtained via conventional enzyme-linked immunosorbent assay (ELISA), which requires several hours. In addition, the estimated detection limits for the three biomarkers were comparable to those of commercially available ELISA kits. Thus, the proposed and fabricated devices facilitate high-precision and frequent monitoring of renal function.

## 1. Introduction

Patients diagnosed with diabetes continue to increase globally owing to changes in social environments and lifestyle habits. Thus, this disease must be focused upon to aid in the development of effective health measures. The International Diabetes Federation, in 2019, stated that the global diabetes population, currently comprising 463 million people, is expected to increase to 700 million by 2045 [1]. A similar increasing trend has been observed in Japan, where, in 2016, approximately 10 million patients were suffering from diabetes, which is a 112% increase over that in 2007 [2].

With the progression of diabetes, a gradual decrease in renal function is inevitable, resulting in diabetic nephropathy. In Japan, diabetic nephropathy is the primary cause of end-stage renal failure, accounting for 43.7% of new dialysis patients [3]. Japan has the largest (per population) number of dialysis patients among the major countries in the world, amounting to approximately 334,000 people [4].

Patients with diabetic nephropathy are required to be admitted to a hospital several times a week to undergo dialysis, imposing enormous stress on the patients. Further, the annual medical expense to manage diabetes is approximately JPY 1.2 trillion, which is equivalent to approximately 4.4% of the annual total medical expenses [5]. Moreover, the annual medical expenditure for dialysis may approximately reach JPY 1.57 trillion, which is a significant problem from the perspective of the medical economic policy of Japan [3]. Therefore, continuous monitoring and relief of the progression of renal injury in patients with diabetes is vital.

Consequently, the quantification of biomarkers in body floods is clinically useful. With the progression of diabetic neuropathy, there is an increase in the concentrations of monocyte chemotactic protein 1 (MCP-1), angiotensinogen (AGT), and liver-type fatty acid-binding protein (L-FABP) [6,7,8,9]. However, the methods currently employed to measure these biomarkers, including microtiter plate-based enzyme-linked immunosorbent assay (ELISA), chemiluminescent enzyme immunoassay, among others require a long assay time and have high costs. Microfabrication technologies can potentially overcome these problems. Diagnostic microdevices offer advantages that can inherently reduce the amount of sample and reagents required (i.e., low invasiveness and cost) and the turnaround time. Moreover, such features allow frequent check-ups, resulting in high-precision monitoring and early diagnosis.

To achieve this, we developed immunoassay devices referred to as immuno-pillar and immuno-wall devices [10,11]. In this study, the ability of immuno-pillar devices to perform high-throughput and highly sensitive immunoassays of urine samples obtained from patients with diabetes was demonstrated. To the best of our knowledge, no experiments on the detection of these three biomarkers from urine samples using microdevices have been conducted. We demonstrated the quantitative detection of the three biomarkers for the progression of diabetic nephropathy in both standard solutions and urine samples. In addition, the accuracy of the quantitative analysis of biomarkers performed using immuno-pillar devices was verified through a comparison with ELISA.

## 2. Materials and Methods

**Figure 1:**
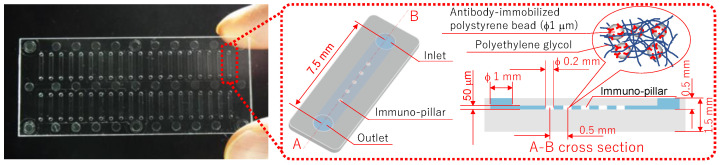
Immuno-pillar device.

Figure 1 shows the immuno-pillar devices that were used to analyze the biomarkers of diabetic nephropathy. Forty straight microchannels were fabricated in a substrate (cyclo olefin polymer), and five porous hydrogel pillars (immuno-pillar) were placed in each microchannel. Moreover, although antibody-immobilized polystyrene beads (1 μm in diameter, Polysciences Inc., Warrington, PA, USA) were physically enclosed within the immuno-pillars, it was possible for the antigen and secondary antibody molecules to reach the polystyrene beads via the pores. Further, sandwich immunoassay procedures were conducted on the surface of the microbeads, to quantitatively detect the antigen molecules, with a detailed description provided later. The fabrication procedures for the immuno-pillar devices were as follows (detailed explanation provided in Ref. [10]). First, polystyrene beads were washed with PBS (pH 7.4, Wako Pure Chemical Industries, Ltd., Osaka, Japan) prior to the preparation of antibody-immobilized polystyrene beads. Thereafter, anti-human MCP-1 monoclonal antibody (R&D Systems, Inc., Minneapolis, MN, USA) was immobilized onto polystyrene beads via hydrophobic interactions. Further, the gap between the immobilized antibodies was coated with BSA (Pierce, Rockford, IL, USA) by immersion in 3% BSA for at least 1 h, to prevent nonspecific binding of the other proteins. Subsequently, the same procedures were individually repeated using anti-AGT monoclonal (Abnova Co., Taipei City, Taiwan) and anti-L-FABP monoclonal (Abcam plc, Cambridge, UK) antibodies.

A vortex mixer was used to stir a mixed solution of PEG-based photocrosslinkable prepolymer MI-1 (50 μL, Kansai Paint Co.,Ltd., Osaka, Japan), photo-initiator PIR-1 (5 μL, Kansai Paint Co.,Ltd., Osaka, Japan), PBS (125 μL), and anti-human MCP-1 monoclonal antibody-immobilized polystyrene bead emulsion (180 μL). Thereafter, this solution was introduced into the microchannels via a pipette (PIPETMAN, Gilson S.A.S., Villiers le Bel, France) and was subsequently irradiated with UV light (wavelength: 365 nm) through a photomask. Further, the photo-crosslinkable prepolymer was polymerized in the microchannel considering the pattern of the photomask. The areas that were exposed became hydrogel pillars containing approximately 3 × 10^5^ anti-MCP-1 monoclonal antibody-immobilized polystyrene beads per pillar. Thereafter, the unreacted prepolymer was removed using an aspirator, and a washing buffer (3% BSA) was used to wash the microchannel, which also removed the polystyrene beads that leaked from the hydrogel. Moreover, to avoid loss of activity of the antibody due to drying, the microchannel was filled with washing buffer prior to use. In addition, the immuno-pillar devices for AGT and L-FABP were also fabricated in a similar manner, as mentioned above.

**Figure 2:**
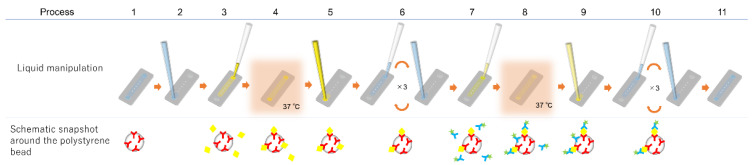
Immunoassay procedure.

The immunoassay procedure using the immuno-pillar device is shown in Figure 2. The same procedure was followed for each biomarker. The injection and removal of the solutions (urine samples, reagents, and washing buffers) were performed using the pipette and aspirator, respectively. Further, the Alexa Fluor 488 Labeling Kit (Invitrogen, Tokyo, Japan) was used to prepare fluorescence-labeled secondary antibody immediately before the immunoassay. First, the washing buffer was removed from the microchannel (step 2) followed by the introduction of 0.25 μL of analyte solution (standard dilution solutions with known concentrations of antigen or patient urine, step 3). This was incubated for 30 s at 37 °C (step 4). Standard dilution solutions of each antigen were prepared in PBS to generate calibration curves. Following the incubation, the solution within the microchannel was removed (step 5), and the microchannel was washed three times with PBS (step 6). Thereafter, 0.25 μL of the fluorescence-labeled secondary antibody was introduced into the microchannel (step 7) and incubated for 30 s at 37 °C (step 8). Subsequently, the solution inside the microchannel was sucked (step 9) followed by washing of the immuno-pillars thrice with PBS again (step 10). Finally, the microchannel was filled with PBS (step 11) and fluorescence images of each immuno-pillar were captured using an inverted fluorescence microscope (Ti-U, Nikon, Japan) equipped with a CCD camera (EM-CCD, Hamamatsu Photonics, Japan) and a super-high-pressure mercury lamp (Nikon, Japan). Consequently, the fluorescence intensity of each immuno-pillar was calculated using the ImageJ software (NIH, Bethesda, MD, USA).

The evaluation of performance and reliability of the immuno-pillar devices was conducted by performing ELISA on the same urine samples using commercial kits (L-FABP: cat. no. 17777, Human L-FABP ELISA Kit, IBL, AGT: cat. no. 27412, Human Total Angiotensinogen Assay Kit, IBL, MCP-1: cat. no. DCP00, Human CCL2/MCP-1 Quantikine ELISA Kit, R&D Systems) and a plate reader. In addition, the antibody reagents used in these kits were the same as those used in the immunoassays of our devices.

The human study was conducted on receiving approval by the Nagoya University Hospital ethical committee for clinical investigation (approval number: 1135), and all participants were provided written informed consent prior to participation. Twenty-six urine samples were obtained at the Nagoya University Hospital from patients categorized into seven stages (0, 1, 2, 3A, 3B, 4, and unknown) of diabetic nephropathy. Further, all urine samples were centrifuged for 5 min (3000× *g*, 4 °C) to remove sediments. Subsequently, the supernatant of each urine sample was divided into two tubes and frozen at −80 °C until they were used in the ELISA or immuno-pillar assay. In addition, the ELISA was performed twice for each urine sample, and the average concentrations estimated using the ELISA are summarized according to the stage of diabetic nephropathy in Table S1.

## 3. Results and Discussion

The concentrations of the three biomarkers of diabetic nephropathy in patient urine samples were estimated by adopting the external standard method. The standard curve for each biomarker was obtained using the standard dilution solutions. The concentration ranges of the standard dilution solutions were 0–100 (MCP-1), 0–200 (AGT), and 0–150 (L-FABP) ng/mL. Further, PBS was used as the dilution factor in the standard dilution solution.

**Figure 3:**
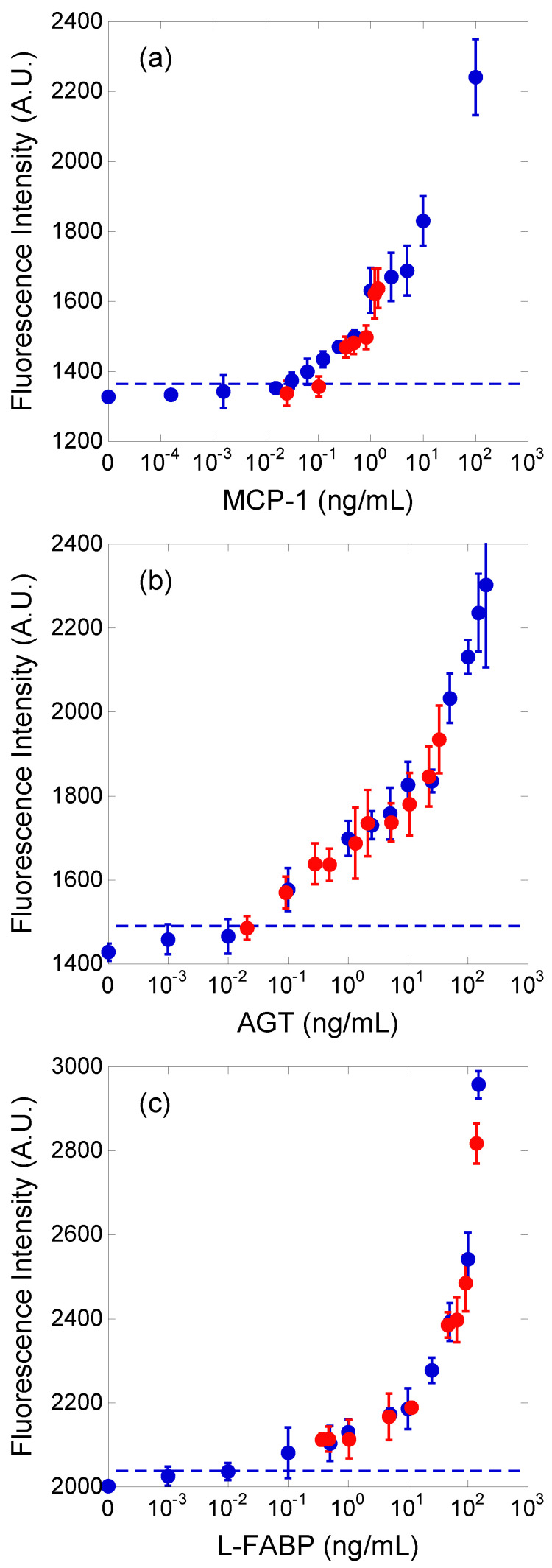
Calibration curves of (**a**) MCP-1, (**b**) AGT, and (**c**) L-FABP. The dashed line in each figure indicates the detection limit. The blue and red plots were obtained with standard dilution solutions and human urine samples, respectively.

The immunoassay procedure (Figure 2) was performed thrice for each standard dilution solution and urine sample. The fluorescence intensities of the immuno-pillar devices obtained after the immunoassays are shown in Figure 3. The blue and red plots correspond to standard dilution solutions and urine samples, respectively. Thereafter, the fluorescence intensity and its standard deviation were calculated from the fluorescence images of 15 immuno-pillars (i.e., three tests using the immuno-pillar devices). The horizontal axis of Figure 3 for the blue plots represents the concentration of the standard dilution solutions that we prepared. In contrast, the concentration of the red plots was determined via the ELISA. As evident, the red plots are close to each calibration curve. The detection limits, which achieved a signal at three standard deviations above the background, were estimated to be 15 (MCP-1), 30 (AGT), and 20 (L-FABP) pg/mL, respectively. Immuno-pillar devices exhibited inherent rapidity during the assay time, which can be attributed to the higher surface-to-volume ratio of the immuno-pillar devices compared to that of the microtiter plates. The total surface area of the immuno-pillar device was approximately 4.7 mm^2^, while the volumes of analytes were 0.25 μL; thus, the surface-to-volume ratio of the immuno-pillar device was 18.8 mm^−1^. In contrast, when considering a commercial flat-bottom 96 ELISA plate (top and bottom diameter: 7 mm, sample volume: 100 μL), the surface area for immunoreaction in a well of the microtiter plate was determined to be approximately 95.6 mm^2^, while the surface-to-volume ratio was 0.956 mm^−1^. Thus, the surface-to-volume ratio of the immuno-pillar device was approximately 20 times larger than that of the microtiter plate. The immobilized antibodies in the immuno-pillar devices more efficiently captured the antigens, resulting in a rapid assay.

The fluorescence intensity obtained from the immuno-pillar devices appeared to exhibit a large deviation, which is attributed to the sponge-like porous structure of the immuno-pillar devices. This microscopic structure renders removal of unbound fluorescence-labeled antibody molecules inside the immuno-pillars a challenging task. In addition, small contaminants, such as cell debris, also cause the decay of analysis precision as they cling closely to polystyrene microbeads and inhibit the antigen–antibody reaction.

**Figure 4:**
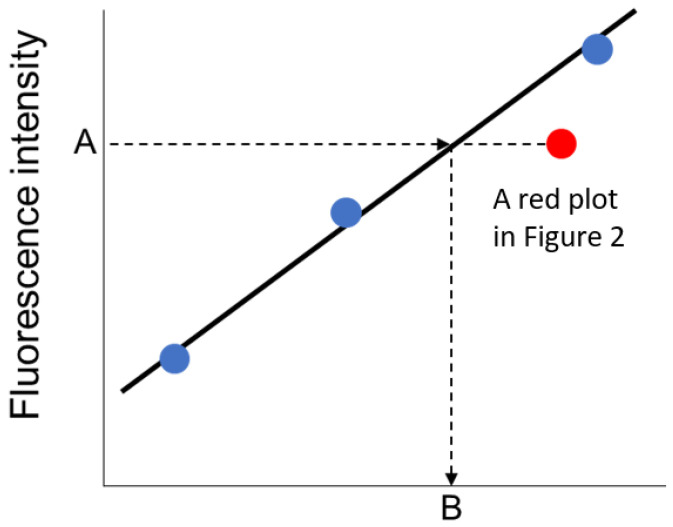
Schematic of the external standard method. Blue plots indicate nearest three data obtained by immuno-pillar devices and standard dilution solutions. (A: fluorescence intensity of immuno-pillar devices; B: concentration estimated by immuno-pillar devices).

To estimate the concentrations of biomarkers in the urine samples, the fluorescence intensity of immuno-pillar devices were analyzed using the external standard method (Figure 4). The least squares line was drawn using the three nearest plots of standard dilution solutions. In the case of the urine sample, the concentration of biomarkers was derived from the least squares line and the average fluorescence intensity of the immuno-pillars, as shown in Figure 4.

**Figure 5:**
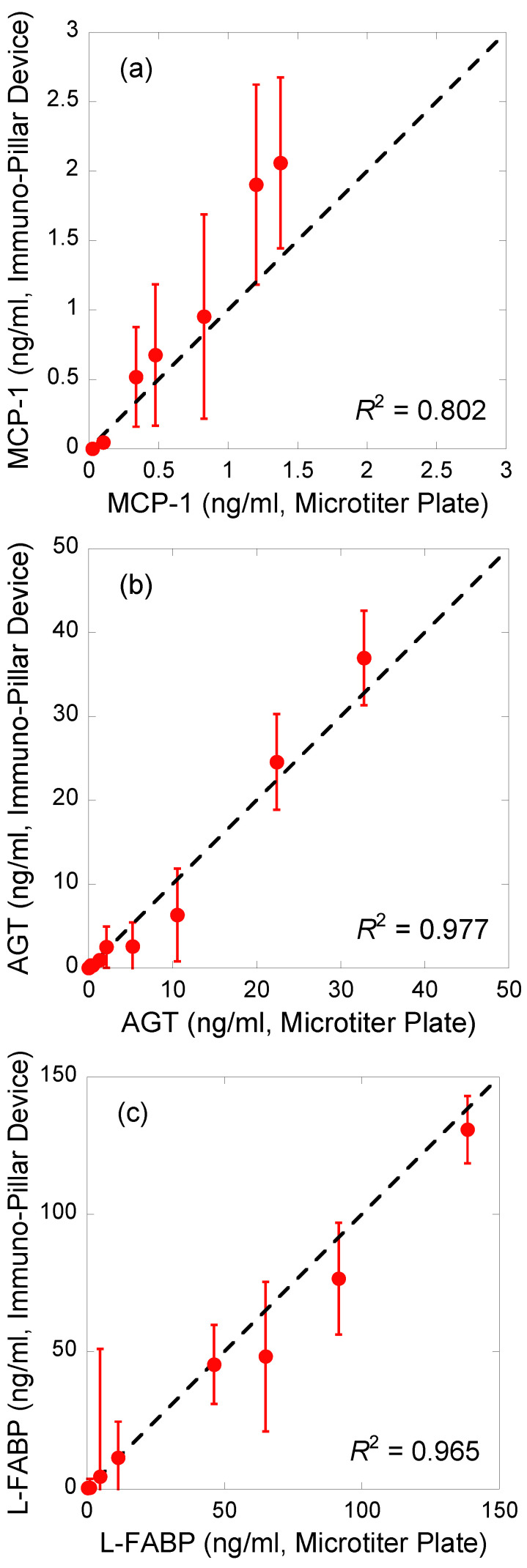
Regression analysis of the correlations between the results obtained using immuno-pillar devices and the conventional ELISA using microtiter plate (**a**) MCP-1, (**b**) AGT, and (**c**) L-FABP. Dashed lines indicate the ideal correlation. The values of coefficient of determination (*R*^2^) denote the correlation between the plots and the dashed line.

Regression analysis of the correlations between the results obtained using the immuno-pillar devices and the conventional ELISA using microtiter plates was conducted to verify the reliability of biomarker concentrations in the urine samples obtained by the immuno-pillar devices (Figure 5). The results obtained using the two were consistent. MCP-1 sensing appeared to show worse performance than the others. We suppose that there are two reasons for this bad accuracy: the concentration range of MCP-1 in the clinical samples and the smoothness of the calibration curve. As shown in Figure 3, MCP-1 concentrations of clinical samples were relatively lower than those of the other markers. It is difficult to analyze such samples with high precision. In addition, the calibration curve was rough between 1 to 10 ng/mL. This also prevents the estimation by using the external standard method. Therefore, we conclude that the clinical potential and urine analysis ability of the immuno-pillar devices were confirmed successfully.

## 4. Conclusions

In this study, we analyzed clinical urine samples obtained from patients with diabetic nephropathy using immuno-pillar devices. The biomarkers of diabetic nephropathy (MCP-1, AGT, and L-FABP) in standard solutions and urine were quantitatively detected in 2 min. Further, we obtained a calibration curve for each biomarker and plotted the data of urine samples on each curve. The urine sample data were consistent with each calibration curve. Finally, to evaluate the correlations between the urine sample data obtained using immuno-pillar devices with those using commercial ELISA kits, regression analysis was performed. The coefficients of determination were estimated to be 0.802 (MCP-1), 0.977 (AGT), and 0.965 (L-FABP). The results indicated consistency between the results of immuno-pillar devices and ELISA kits, implying the availability of immuno-pillar devices for the rapid analysis of clinical urine samples.

## Supplementary Materials

The following is available online at www.mdpi.com/article/10.3390/mi12111353/s1, Table S1: Patient characteristics and immunoassay results.
