# Supplementary material for: Microchip Immunoassays for Monitoring Renal Function: Rapid, Low-Cost, and Highly Sensitive Quantification of Urinary Biomarkers of Diabetic Nephropathy
Source: Micromachines (Basel). 2021 Oct 31;12(11):1353. doi: 10.3390/mi12111353 (PMC8621389; doi:10.3390/mi12111353)
Supplement: Supplementary file 1 [file micromachines-12-01353-s001.zip › micromachines-1419812-supplementary.pdf]

Microchip Immunoassays for Monitoring Renal Function: Rapid, Low-Cost, and Highly Sensitive Quantification of Urinary Biomarkers of Diabetic Nephropathy

Toshihiro Kasama [1,2,*], Miaomiao Sun [3], Noritada Kaji [2,4], Shin-ichi Akiyama [5], Yukio Yuzawa [6], Manabu Tokeshi [2,7], Seiichi Matsuo [5] and Yoshinobu Baba [2,3,8]

[1] Department of Bioengineering, Graduate School of Engineering, The University of Tokyo, Tokyo 113-8654, Japan
[2] Institute of Nano-Life-Systems, Institutes of Innovation for Future Society, Nagoya University, Aichi 464-8603, Japan
[3] Department of Biomolecular Engineering, Graduate School of Engineering, Nagoya University, Aichi 464-8603, Japan
[4] Department of Applied Chemistry, Faculty of Engineering, Kyushu University, Fukuoka 819-0395, Japan
[5] Division of Nephrology, Department of Internal Medicine, Nagoya University Graduate School of Medicine, Aichi 466-8550, Japan
[6] Department of Nephrology, Fujita Health University, Aichi 470-1192, Japan
[7] Division of Applied Chemistry, Faculty of Engineering, Hokkaido University, Hokkaido 060-0808, Japan
[8] Health Research Institute, National Institute of Advanced Industrial Science and Technology, Kagawa 761- 0395, Japan
* Correspondence: kasama.toshihiro@kk.alumni.u-tokyo.ac.jp

Table S1. Patient characteristics and immunoassay results.

No.	Age	Sex	Stage of diabetic nephropathy	Concentration of MCP-1 (ng/mL)	
				ELISA	Immuno-pillar analysis
1	72	M	4	1.38	2.06
2	53	M	4	0.83	0.95
3	65	F	3A	1.20	1.90
4	82	F	3A	0.34	0.52
5	71	M	2	0.48	0.68
6	58	M	0	0.10	0.05
7	32	F	0	0.03	0.001

No.	Age	Sex	Stage of diabetic nephropathy	Concentration of AGT (ng/mL)	
				ELISA	Immuno-pillar analysis
8	69	M	4	32.8	37.0
9	53	M	4	22.4	24.6
10	68	F	3A	10.6	6.33
11	65	F	3A	5.25	2.58
12	71	M	2	0.28	0.34
13	48	F	1	1.32	0.94

14	55	F	1	0.09	0.08
15	69	F	1	0.02	0.02
16	58	F	0	0.49	0.33
17	58	M	0	2.12	2.50

No.	Age	Sex	Stage of diabetic nephropathy	Concentration of L-FABP (ng/mL)	
				ELISA	Immuno-pillar analysis
18	72	M	4	138.5	130.8
19	69	M	4	91.5	76.5
20	53	M	4	65.0	48.3
21	77	F	4	46.2	45.4
22	72	M	3A	11.4	11.4
23	63	M	2	1.04	0.50
24	61	M	1	4.81	4.65
25	48	F	1	0.37	0.48
26	51	F	0	0.48	0.50
